# 4-[(*E*)-(4-Diethyl­amino-2-hy­droxy­benzyl­idene)amino]-1,5-dimethyl-2-phenyl-1*H*-pyrazol-3(2*H*)-one

**DOI:** 10.1107/S1600536811037615

**Published:** 2011-09-20

**Authors:** K. Manvizhi, G. Chakkaravarthi, G. Anbalagan, G. Rajagopal

**Affiliations:** a Department of Chemistry, Anand Institute of Higher Technology, Kazhipattur, Chennai 603 103, India; b Department of Physics, CPCL Polytechnic College, Chennai 600 068, India; c Department of Physics, Presidency College (Autonomous), Chennai 600 005, India; d Department of Chemistry, Government Arts College, Melur 625 106, India

## Abstract

In the title compound, C_22_H_26_N_4_O_2_, the phenyl ring and hy­droxy­benzene group are twisted with respect to the central pyrazolone ring, making dihedral angles of 54.05 (5) and 21.80 (6)°, respectively. One of the ethyl groups is disordered over two positions with site occupancies of 0.872 (6) and 0.128 (6). The mol­ecular structure features short intra­molecular O—H⋯N and C—H⋯O contacts. The crystal packing exhibits weak inter­molecular C—H⋯O and C—H⋯π inter­actions.

## Related literature

For biological activities of pyrazolone derivatives, see: Gursoy *et al.* (2000 ▶); Ragavan *et al.* (2009 ▶). For related structures, see: Wang *et al.* (2007 ▶); Zhu *et al.* (2008 ▶).
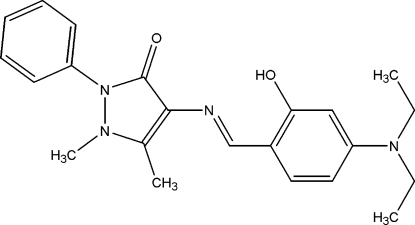


## Experimental

### 

#### Crystal data

C_22_H_26_N_4_O_2_
*M* *_r_* = 378.47
Monoclinic, 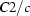

*a* = 17.2794 (6) Å
*b* = 7.1853 (3) Å
*c* = 32.9711 (12) Å
β = 101.652 (1)°
*V* = 4009.3 (3) Å^3^
*Z* = 8
Mo *K*α radiation
μ = 0.08 mm^−1^
*T* = 295 K
0.28 × 0.24 × 0.20 mm

#### Data collection

Bruker Kappa APEXII diffractometer
Absorption correction: multi-scan (*SADABS*; Sheldrick, 1996 ▶) *T*_min_ = 0.977, *T*_max_ = 0.984
21210 measured reflections
4398 independent reflections
3293 reflections with *I* > 2σ(*I*)
*R*_int_ = 0.029

#### Refinement

*R*[*F* ^2^ > 2σ(*F* ^2^)] = 0.049
*wR*(*F* ^2^) = 0.126
*S* = 1.03
4398 reflections
274 parameters
3 restraints
H-atom parameters constrained
Δρ_max_ = 0.33 e Å^−3^
Δρ_min_ = −0.18 e Å^−3^

### 

Data collection: *APEX2* (Bruker, 2004 ▶); cell refinement: *SAINT* (Bruker, 2004 ▶); data reduction: *SAINT*; program(s) used to solve structure: *SHELXS97* (Sheldrick, 2008 ▶); program(s) used to refine structure: *SHELXL97* (Sheldrick, 2008 ▶); molecular graphics: *PLATON* (Spek, 2009 ▶); software used to prepare material for publication: *SHELXL97*.

## Supplementary Material

Crystal structure: contains datablock(s) global, I. DOI: 10.1107/S1600536811037615/gk2400sup1.cif

Structure factors: contains datablock(s) I. DOI: 10.1107/S1600536811037615/gk2400Isup2.hkl

Supplementary material file. DOI: 10.1107/S1600536811037615/gk2400Isup3.cml

Additional supplementary materials:  crystallographic information; 3D view; checkCIF report

## Figures and Tables

**Table 1:** Hydrogen-bond geometry (Å, °) *Cg*1 and *Cg*2 are the centroids of the N1/N2/C7/C8/C9 and C1–C6 rings, respectively.

*D*—H⋯*A*	*D*—H	H⋯*A*	*D*⋯*A*	*D*—H⋯*A*
O2—H2*A*⋯N3	0.82	1.88	2.6127 (17)	148
C12—H12⋯O1	0.93	2.33	3.004 (2)	130
C2—H2⋯O2^i^	0.93	2.48	3.267 (2)	142
C11—H11*B*⋯O1^ii^	0.96	2.36	3.309 (2)	172
C4—H4⋯*Cg*2^iii^	0.93	2.89	3.746 (2)	153
C6—H6⋯*Cg*1^iv^	0.93	2.78	3.559 (2)	142

Symmetry codes: (i) 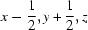; (ii) 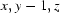; (iii) 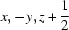; (iv) 
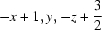
.

**Fig. 1.**
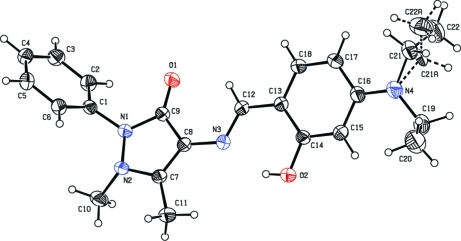
The molecular structure of the title compound and 30% probability displacement ellipsoids for non-H atoms.

**Fig. 2.**
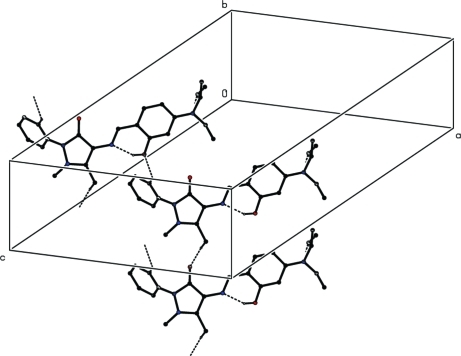
The crystal packing of the title compound. Hydrogen bonds are shown as dashed lines. H atoms not involved in hydrogen bonding have been omitted.

## supplementary crystallographic information

### Comment

Pyrazolone derivatives exhibit antipyretic, anti-inflammatory, antibacterial and antifungal (Gursoy *et al.*, 2000; Ragavan *et al.*, 2009) activities. The geometric parameters of the title compound, (Fig. 1) agree well with the reported similar structures (Wang *et al.*, 2007; Zhu *et al.*, 2008).

The phenyl ring (C1–C6) and hydroxybenzene group (C13–C18) are twisted with respect to the pyrazolone ring (N1/N2/C7/C8/C9), making the dihedral angles of and 54.05 (5) and 21.80 (6)°, respectively. One of the ethyl groups is disordered over two positions with site occupancies of 0.872 (6) and 0.128 (6). The molecular structure is stabilized by weak intramolecular O—H···N and C—H···O interactions and the crystal packing exhibits weak intermolecular C—H···O and C—H···π  interactions (Table 1 & Fig. 2).

### Experimental

A solution of 1-phenyl-2,3-dimethyl-4-amino-3-pyrazolin-5-one (0.203 g, 1 mmol) in ethanol (5 ml) was added to a solution of 4-diethylamino-2-hydroxybenzaldehyde (0.193 g, 1 mmol) in ethanol (5 ml). The reaction mixture was stirred for 2 h at room temperature then heated to reflux for 2 h and kept at 273 K for 4h. The characteristic pale-green precipitate obtained was filtered and recrystallized by dissolving in methanol (m.p. 438 K). Single crystals suitable for X-ray diffraction were obtained by slow evaporation of a solution of the title compound in methanol at room temperature.

### Refinement

The site occupancy factors for disordered C atoms of one of the ethyl groups refined at: C21 sof = 0.872 (6), C22 sof = 0.872 (6), C21A sof = 0.128 (6), C22A sof = 0.128 (6). The bond distances N4—C21A and C21A—C22A were restrained to 1.48 (1) Å and 1.54 (1) Å, respectively and the non-bonding distance N4—C22A was restrained to 2.34 (1) Å. All H atoms were positioned geometrically with C—H = 0.93–0.97 Å and O–H = 0.82 Å and allowed to ride on their parent atoms, with U_iso_(H) = 1.5U_eq_(O) or 1.2U_eq_(C) or 1.5U_eq_(C_methyl_).

### Crystal data

**Table:**

C_22_H_26_N_4_O_2_	*F*(000) = 1616
*M**_r_* = 378.47	*D*_x_ = 1.254 Mg m^−^^3^
Monoclinic, *C*2/*c*	Mo *K*α radiation, λ = 0.71073 Å
Hall symbol: -C 2yc	Cell parameters from 22393 reflections
*a* = 17.2794 (6) Å	θ = 2.4–27.1°
*b* = 7.1853 (3) Å	µ = 0.08 mm^−^^1^
*c* = 32.9711 (12) Å	*T* = 295 K
β = 101.652 (1)°	Block, colourless
*V* = 4009.3 (3) Å^3^	0.28 × 0.24 × 0.20 mm
*Z* = 8	

### Data collection

**Table:**

Bruker Kappa APEXII diffractometer	4398 independent reflections
Radiation source: fine-focus sealed tube	3293 reflections with *I* > 2σ(*I*)
graphite	*R*_int_ = 0.029
ω and φ scans	θ_max_ = 27.1°, θ_min_ = 2.4°
Absorption correction: multi-scan (*SADABS*; Sheldrick, 1996)	*h* = −18→22
*T*_min_ = 0.977, *T*_max_ = 0.984	*k* = −8→9
21210 measured reflections	*l* = −42→41

### Refinement

**Table:**

Refinement on *F*^2^	Primary atom site location: structure-invariant direct methods
Least-squares matrix: full	Secondary atom site location: difference Fourier map
*R*[*F*^2^ > 2σ(*F*^2^)] = 0.049	Hydrogen site location: inferred from neighbouring sites
*wR*(*F*^2^) = 0.126	H-atom parameters constrained
*S* = 1.03	*w* = 1/[σ^2^(*F*_o_^2^) + (0.052*P*)^2^ + 2.4774*P*] where *P* = (*F*_o_^2^ + 2*F*_c_^2^)/3
4398 reflections	(Δ/σ)_max_ < 0.001
274 parameters	Δρ_max_ = 0.33 e Å^−^^3^
3 restraints	Δρ_min_ = −0.18 e Å^−^^3^

### Fractional atomic coordinates and isotropic or equivalent isotropic displacement parameters (Å^2^)

**Table:**

	*x*	*y*	*z*	*U*_iso_*/*U*_eq_	Occ. (<1)
O1	0.45300 (7)	0.35172 (17)	0.64038 (4)	0.0550 (3)	
O2	0.70119 (7)	−0.04760 (16)	0.59423 (4)	0.0509 (3)	
H2A	0.6621	−0.0508	0.6048	0.076*	
N1	0.41671 (8)	0.09879 (18)	0.67543 (4)	0.0421 (3)	
N2	0.45087 (8)	−0.07360 (18)	0.68900 (4)	0.0427 (3)	
N3	0.58759 (8)	0.08183 (19)	0.62786 (4)	0.0415 (3)	
N4	0.87010 (11)	0.3824 (2)	0.54493 (6)	0.0730 (5)	
C1	0.37186 (9)	0.1964 (2)	0.70049 (5)	0.0392 (4)	
C2	0.31096 (10)	0.3083 (2)	0.68109 (5)	0.0473 (4)	
H2	0.2973	0.3121	0.6523	0.057*	
C3	0.27040 (10)	0.4148 (3)	0.70461 (6)	0.0529 (4)	
H3	0.2295	0.4916	0.6916	0.063*	
C4	0.28989 (10)	0.4086 (3)	0.74715 (6)	0.0511 (4)	
H4	0.2628	0.4818	0.7629	0.061*	
C5	0.34974 (10)	0.2935 (3)	0.76613 (5)	0.0505 (4)	
H5	0.3625	0.2877	0.7949	0.061*	
C6	0.39114 (10)	0.1864 (2)	0.74313 (5)	0.0456 (4)	
H6	0.4315	0.1085	0.7562	0.055*	
C7	0.51277 (9)	−0.0988 (2)	0.66911 (5)	0.0403 (4)	
C8	0.52434 (9)	0.0572 (2)	0.64794 (5)	0.0396 (4)	
C9	0.46392 (9)	0.1904 (2)	0.65229 (5)	0.0404 (4)	
C10	0.39353 (12)	−0.2229 (3)	0.69031 (7)	0.0615 (5)	
H10A	0.3620	−0.2414	0.6631	0.092*	
H10B	0.3600	−0.1891	0.7090	0.092*	
H10C	0.4211	−0.3359	0.6996	0.092*	
C11	0.55860 (11)	−0.2746 (2)	0.67399 (6)	0.0568 (5)	
H11A	0.6023	−0.2638	0.6602	0.085*	
H11B	0.5251	−0.3753	0.6621	0.085*	
H11C	0.5781	−0.2985	0.7029	0.085*	
C12	0.60614 (10)	0.2457 (2)	0.61670 (5)	0.0443 (4)	
H12	0.5753	0.3467	0.6212	0.053*	
C13	0.67255 (10)	0.2769 (2)	0.59774 (5)	0.0423 (4)	
C14	0.71893 (10)	0.1312 (2)	0.58723 (5)	0.0413 (4)	
C15	0.78306 (11)	0.1657 (2)	0.56953 (6)	0.0518 (4)	
H15	0.8120	0.0662	0.5624	0.062*	
C16	0.80566 (11)	0.3479 (3)	0.56208 (6)	0.0534 (5)	
C17	0.75989 (12)	0.4944 (3)	0.57316 (6)	0.0572 (5)	
H17	0.7736	0.6170	0.5690	0.069*	
C18	0.69576 (11)	0.4578 (2)	0.58988 (6)	0.0544 (5)	
H18	0.6660	0.5572	0.5964	0.065*	
C19	0.90916 (13)	0.2322 (3)	0.52606 (7)	0.0717 (6)	
H19A	0.9297	0.2818	0.5030	0.086*	
H19B	0.8708	0.1365	0.5155	0.086*	
C20	0.97484 (16)	0.1490 (4)	0.55681 (9)	0.0939 (8)	
H20A	1.0114	0.2448	0.5684	0.141*	
H20B	1.0016	0.0576	0.5434	0.141*	
H20C	0.9539	0.0908	0.5785	0.141*	
C21	0.90416 (14)	0.5704 (4)	0.54439 (8)	0.0585 (8)	0.872 (6)
H21A	0.9610	0.5611	0.5474	0.070*	0.872 (6)
H21B	0.8932	0.6419	0.5675	0.070*	0.872 (6)
C22	0.87019 (19)	0.6673 (5)	0.50495 (9)	0.0881 (11)	0.872 (6)
H22A	0.8930	0.7892	0.5051	0.132*	0.872 (6)
H22B	0.8140	0.6781	0.5022	0.132*	0.872 (6)
H22C	0.8816	0.5972	0.4821	0.132*	0.872 (6)
C21A	0.8615 (8)	0.5403 (16)	0.5143 (3)	0.051 (5)	0.128 (6)
H21C	0.8079	0.5875	0.5079	0.062*	0.128 (6)
H21D	0.8775	0.5039	0.4889	0.062*	0.128 (6)
C22A	0.9190 (13)	0.6828 (18)	0.5389 (6)	0.092 (8)	0.128 (6)
H22D	0.9194	0.7934	0.5226	0.139*	0.128 (6)
H22E	0.9712	0.6306	0.5453	0.139*	0.128 (6)
H22F	0.9022	0.7133	0.5641	0.139*	0.128 (6)

### Atomic displacement parameters (Å^2^)

**Table:**

	*U*^11^	*U*^22^	*U*^33^	*U*^12^	*U*^13^	*U*^23^
O1	0.0649 (8)	0.0415 (7)	0.0644 (8)	0.0090 (6)	0.0270 (6)	0.0139 (6)
O2	0.0591 (7)	0.0380 (6)	0.0599 (8)	0.0027 (5)	0.0222 (6)	0.0033 (5)
N1	0.0471 (7)	0.0356 (7)	0.0455 (8)	0.0029 (6)	0.0140 (6)	0.0042 (6)
N2	0.0473 (8)	0.0321 (7)	0.0508 (8)	−0.0009 (6)	0.0152 (6)	0.0029 (6)
N3	0.0456 (7)	0.0420 (8)	0.0383 (7)	−0.0003 (6)	0.0116 (6)	−0.0009 (6)
N4	0.0753 (11)	0.0561 (10)	0.1027 (14)	0.0042 (9)	0.0540 (11)	0.0114 (10)
C1	0.0371 (8)	0.0373 (8)	0.0447 (9)	−0.0034 (6)	0.0117 (7)	0.0018 (7)
C2	0.0460 (9)	0.0501 (10)	0.0442 (9)	0.0038 (8)	0.0057 (7)	0.0050 (8)
C3	0.0444 (9)	0.0506 (10)	0.0637 (12)	0.0102 (8)	0.0111 (8)	0.0072 (9)
C4	0.0493 (10)	0.0489 (10)	0.0599 (11)	0.0029 (8)	0.0220 (8)	−0.0030 (8)
C5	0.0510 (10)	0.0596 (11)	0.0423 (9)	0.0001 (9)	0.0127 (8)	0.0000 (8)
C6	0.0403 (8)	0.0505 (10)	0.0463 (9)	0.0049 (7)	0.0094 (7)	0.0075 (8)
C7	0.0443 (9)	0.0355 (8)	0.0408 (8)	−0.0031 (7)	0.0079 (7)	−0.0049 (7)
C8	0.0443 (8)	0.0380 (8)	0.0367 (8)	−0.0015 (7)	0.0089 (7)	−0.0033 (7)
C9	0.0456 (9)	0.0374 (9)	0.0391 (8)	−0.0009 (7)	0.0107 (7)	0.0025 (7)
C10	0.0621 (11)	0.0438 (10)	0.0835 (14)	−0.0112 (9)	0.0260 (10)	0.0030 (10)
C11	0.0640 (11)	0.0408 (10)	0.0684 (12)	0.0062 (8)	0.0200 (10)	0.0021 (9)
C12	0.0506 (9)	0.0395 (9)	0.0453 (9)	0.0040 (7)	0.0155 (8)	−0.0003 (7)
C13	0.0485 (9)	0.0392 (9)	0.0416 (9)	0.0032 (7)	0.0146 (7)	0.0028 (7)
C14	0.0499 (9)	0.0366 (9)	0.0373 (8)	0.0019 (7)	0.0086 (7)	0.0034 (7)
C15	0.0548 (10)	0.0448 (10)	0.0614 (11)	0.0085 (8)	0.0251 (9)	0.0027 (8)
C16	0.0563 (10)	0.0502 (10)	0.0591 (11)	0.0029 (8)	0.0244 (9)	0.0083 (9)
C17	0.0679 (12)	0.0398 (10)	0.0710 (12)	−0.0001 (9)	0.0310 (10)	0.0076 (9)
C18	0.0639 (11)	0.0381 (9)	0.0681 (12)	0.0055 (8)	0.0298 (10)	0.0035 (8)
C19	0.0736 (14)	0.0761 (15)	0.0746 (14)	0.0043 (12)	0.0364 (12)	0.0106 (12)
C20	0.0863 (17)	0.098 (2)	0.102 (2)	0.0101 (15)	0.0298 (15)	0.0236 (16)
C21	0.0556 (14)	0.0675 (18)	0.0537 (15)	−0.0115 (12)	0.0141 (12)	0.0029 (13)
C22	0.103 (2)	0.093 (3)	0.0670 (18)	−0.0082 (19)	0.0142 (17)	0.0196 (17)
C21A	0.066 (10)	0.051 (10)	0.045 (9)	−0.005 (7)	0.028 (8)	0.015 (7)
C22A	0.109 (17)	0.061 (14)	0.105 (19)	−0.019 (12)	0.016 (14)	−0.003 (13)

### Geometric parameters (Å, °)

**Table:**

O1—C9	1.2260 (19)	C11—H11B	0.9600
O2—C14	1.3514 (19)	C11—H11C	0.9600
O2—H2A	0.8200	C12—C13	1.431 (2)
N1—C9	1.390 (2)	C12—H12	0.9300
N1—N2	1.4058 (18)	C13—C18	1.400 (2)
N1—C1	1.426 (2)	C13—C14	1.404 (2)
N2—C7	1.375 (2)	C14—C15	1.375 (2)
N2—C10	1.467 (2)	C15—C16	1.402 (3)
N3—C12	1.293 (2)	C15—H15	0.9300
N3—C8	1.399 (2)	C16—C17	1.408 (3)
N4—C16	1.369 (2)	C17—C18	1.359 (2)
N4—C21	1.475 (3)	C17—H17	0.9300
N4—C19	1.475 (3)	C18—H18	0.9300
N4—C21A	1.506 (9)	C19—C20	1.486 (3)
C1—C2	1.375 (2)	C19—H19A	0.9700
C1—C6	1.380 (2)	C19—H19B	0.9700
C2—C3	1.378 (2)	C20—H20A	0.9600
C2—H2	0.9300	C20—H20B	0.9600
C3—C4	1.375 (3)	C20—H20C	0.9600
C3—H3	0.9300	C21—C22	1.487 (4)
C4—C5	1.372 (2)	C21—H21A	0.9700
C4—H4	0.9300	C21—H21B	0.9700
C5—C6	1.378 (2)	C22—H22A	0.9600
C5—H5	0.9300	C22—H22B	0.9600
C6—H6	0.9300	C22—H22C	0.9600
C7—C8	1.357 (2)	C21A—C22A	1.538 (10)
C7—C11	1.482 (2)	C21A—H21C	0.9700
C8—C9	1.445 (2)	C21A—H21D	0.9700
C10—H10A	0.9600	C22A—H22D	0.9600
C10—H10B	0.9600	C22A—H22E	0.9600
C10—H10C	0.9600	C22A—H22F	0.9600
C11—H11A	0.9600		
			
C14—O2—H2A	109.5	C18—C13—C14	116.60 (15)
C9—N1—N2	109.69 (12)	C18—C13—C12	120.76 (15)
C9—N1—C1	122.18 (13)	C14—C13—C12	122.61 (15)
N2—N1—C1	119.43 (12)	O2—C14—C15	118.29 (15)
C7—N2—N1	106.34 (12)	O2—C14—C13	120.43 (14)
C7—N2—C10	120.86 (14)	C15—C14—C13	121.28 (15)
N1—N2—C10	114.28 (13)	C14—C15—C16	121.32 (16)
C12—N3—C8	120.80 (14)	C14—C15—H15	119.3
C16—N4—C21	122.02 (17)	C16—C15—H15	119.3
C16—N4—C19	121.42 (17)	N4—C16—C15	121.31 (17)
C21—N4—C19	116.56 (16)	N4—C16—C17	121.21 (17)
C16—N4—C21A	115.8 (6)	C15—C16—C17	117.47 (16)
C19—N4—C21A	105.1 (5)	C18—C17—C16	120.49 (17)
C2—C1—C6	120.60 (15)	C18—C17—H17	119.8
C2—C1—N1	118.19 (14)	C16—C17—H17	119.8
C6—C1—N1	121.13 (14)	C17—C18—C13	122.82 (16)
C1—C2—C3	119.43 (16)	C17—C18—H18	118.6
C1—C2—H2	120.3	C13—C18—H18	118.6
C3—C2—H2	120.3	N4—C19—C20	111.0 (2)
C4—C3—C2	120.56 (16)	N4—C19—H19A	109.4
C4—C3—H3	119.7	C20—C19—H19A	109.4
C2—C3—H3	119.7	N4—C19—H19B	109.4
C5—C4—C3	119.43 (16)	C20—C19—H19B	109.4
C5—C4—H4	120.3	H19A—C19—H19B	108.0
C3—C4—H4	120.3	C19—C20—H20A	109.5
C4—C5—C6	120.85 (17)	C19—C20—H20B	109.5
C4—C5—H5	119.6	H20A—C20—H20B	109.5
C6—C5—H5	119.6	C19—C20—H20C	109.5
C5—C6—C1	119.11 (16)	H20A—C20—H20C	109.5
C5—C6—H6	120.4	H20B—C20—H20C	109.5
C1—C6—H6	120.4	N4—C21—C22	110.6 (2)
C8—C7—N2	110.26 (14)	N4—C21—H21A	109.5
C8—C7—C11	128.98 (15)	C22—C21—H21A	109.5
N2—C7—C11	120.70 (14)	N4—C21—H21B	109.5
C7—C8—N3	123.96 (15)	C22—C21—H21B	109.5
C7—C8—C9	108.17 (14)	H21A—C21—H21B	108.1
N3—C8—C9	127.78 (14)	C21—C22—H22A	109.5
O1—C9—N1	123.66 (15)	C21—C22—H22B	109.5
O1—C9—C8	131.45 (15)	H22A—C22—H22B	109.5
N1—C9—C8	104.87 (13)	C21—C22—H22C	109.5
N2—C10—H10A	109.5	H22A—C22—H22C	109.5
N2—C10—H10B	109.5	H22B—C22—H22C	109.5
H10A—C10—H10B	109.5	N4—C21A—C22A	100.5 (8)
N2—C10—H10C	109.5	N4—C21A—H21C	111.7
H10A—C10—H10C	109.5	C22A—C21A—H21C	111.7
H10B—C10—H10C	109.5	N4—C21A—H21D	111.7
C7—C11—H11A	109.5	C22A—C21A—H21D	111.7
C7—C11—H11B	109.5	H21C—C21A—H21D	109.4
H11A—C11—H11B	109.5	C21A—C22A—H22D	109.5
C7—C11—H11C	109.5	C21A—C22A—H22E	109.5
H11A—C11—H11C	109.5	H22D—C22A—H22E	109.5
H11B—C11—H11C	109.5	C21A—C22A—H22F	109.5
N3—C12—C13	122.26 (15)	H22D—C22A—H22F	109.5
N3—C12—H12	118.9	H22E—C22A—H22F	109.5
C13—C12—H12	118.9		
			
C9—N1—N2—C7	−8.57 (17)	N3—C8—C9—N1	−178.70 (15)
C1—N1—N2—C7	−156.94 (13)	C8—N3—C12—C13	177.22 (15)
C9—N1—N2—C10	−144.52 (15)	N3—C12—C13—C18	−173.45 (17)
C1—N1—N2—C10	67.11 (19)	N3—C12—C13—C14	4.6 (3)
C9—N1—C1—C2	65.9 (2)	C18—C13—C14—O2	179.23 (16)
N2—N1—C1—C2	−149.84 (15)	C12—C13—C14—O2	1.1 (2)
C9—N1—C1—C6	−111.09 (18)	C18—C13—C14—C15	−1.0 (2)
N2—N1—C1—C6	33.2 (2)	C12—C13—C14—C15	−179.12 (17)
C6—C1—C2—C3	1.7 (3)	O2—C14—C15—C16	−178.86 (17)
N1—C1—C2—C3	−175.26 (15)	C13—C14—C15—C16	1.4 (3)
C1—C2—C3—C4	−0.6 (3)	C21—N4—C16—C15	−167.7 (2)
C2—C3—C4—C5	−0.7 (3)	C19—N4—C16—C15	12.2 (3)
C3—C4—C5—C6	0.9 (3)	C21A—N4—C16—C15	141.5 (6)
C4—C5—C6—C1	0.2 (3)	C21—N4—C16—C17	11.9 (3)
C2—C1—C6—C5	−1.5 (2)	C19—N4—C16—C17	−168.2 (2)
N1—C1—C6—C5	175.37 (15)	C21A—N4—C16—C17	−39.0 (6)
N1—N2—C7—C8	7.11 (18)	C14—C15—C16—N4	179.18 (18)
C10—N2—C7—C8	139.51 (16)	C14—C15—C16—C17	−0.4 (3)
N1—N2—C7—C11	−175.49 (14)	N4—C16—C17—C18	179.5 (2)
C10—N2—C7—C11	−43.1 (2)	C15—C16—C17—C18	−0.9 (3)
N2—C7—C8—N3	173.56 (14)	C16—C17—C18—C13	1.3 (3)
C11—C7—C8—N3	−3.6 (3)	C14—C13—C18—C17	−0.3 (3)
N2—C7—C8—C9	−3.09 (18)	C12—C13—C18—C17	177.84 (18)
C11—C7—C8—C9	179.78 (16)	C16—N4—C19—C20	−92.3 (3)
C12—N3—C8—C7	−163.62 (16)	C21—N4—C19—C20	87.6 (2)
C12—N3—C8—C9	12.3 (2)	C21A—N4—C19—C20	133.9 (6)
N2—N1—C9—O1	−171.84 (16)	C16—N4—C21—C22	−93.5 (3)
C1—N1—C9—O1	−24.5 (2)	C19—N4—C21—C22	86.6 (3)
N2—N1—C9—C8	6.62 (17)	C21A—N4—C21—C22	2.0 (8)
C1—N1—C9—C8	153.96 (14)	C16—N4—C21A—C22A	110.9 (11)
C7—C8—C9—O1	176.07 (18)	C21—N4—C21A—C22A	0.5 (10)
N3—C8—C9—O1	−0.4 (3)	C19—N4—C21A—C22A	−112.3 (11)
C7—C8—C9—N1	−2.22 (18)		

### Hydrogen-bond geometry (Å, °)

**Table:**

Cg1 and Cg2 are the centroids of the N1/N2/C7/C8/C9 and C1–C6 rings, respectively.

**Table:**

*D*—H···*A*	*D*—H	H···*A*	*D*···*A*	*D*—H···*A*
O2—H2A···N3	0.82	1.88	2.6127 (17)	148
C12—H12···O1	0.93	2.33	3.004 (2)	130
C2—H2···O2^i^	0.93	2.48	3.267 (2)	142
C11—H11B···O1^ii^	0.96	2.36	3.309 (2)	172
C4—H4···Cg2^iii^	0.93	2.89	3.746 (2)	153
C6—H6···Cg1^iv^	0.93	2.78	3.559 (2)	142

Symmetry codes: (i) *x*−1/2, *y*+1/2, *z*; (ii) *x*, *y*−1, *z*; (iii) *x*, −*y*, *z*+1/2; (iv) −*x*+1, *y*, −*z*+3/2.
